# Laparoscopic surgery complications: Postoperative peritonitis

**Published:** 2012-09-25

**Authors:** L Drăghici, I Drăghici, A Ungureanu, C Copăescu, M Popescu, C Dragomirescu

**Affiliations:** * Surgery Department of „Sf. Ioan” Clinical Emergency Hospital, Bucharest, Romania; ** Department of Pediatric Surgery, “Maria Sklodowska Curie” Emergency Hospital for Children, Bucharest, Romania; *** Surgery Department of “Delta PRO Medical” Hospital, “Carol Davila” University of Medicine and Pharmacy, Bucharest, Romania

**Keywords:** incidents, celioscopic method, reinterventions, emergencies

## Abstract

**Introduction: **Complications within laparoscopic surgery, similar to classic surgery are inevitable and require immediate actions both to diminish intraoperative risks and to choose the appropriate therapeutic attitude. Peritonitis and hemorrhagic incidents are both part of the complications aspect of laparoscopic surgery. Fortunately, the incidence is limited, thus excluding the rejection of celioscopic methods. Patient’s risks and benefits are to be analyzed carefully prior recommending laparoscopic surgery.

**Materials and methods: **This study presents a statistical analysis of peritonitis consecutive to laparoscopic surgery, experience of „Sf. Ioan” Emergency Hospital, Bucharest, and Department of Surgery (2000-2010).

**Results:**There were 180 (0,96%) complicated situations requiring reinterventions, from a total of 18676 laparoscopic procedures. 106 cases (0,56%) represented different grades of postoperative peritonitis. Most frequently, there were consecutive laparoscopic appendicectomia and colecistectomia. During the last decade, few severe cases of peritonitis followed laparoscopic bariatric surgical procedures.

**Conclusions:** This study reflects the possibility of unfavorable evolution of postoperative peritonitis comparing with hemorrhagic incidents within laparoscopic surgery.

## Introduction

In the initial period of laparoscopic activity, complications are inevitable, thus the risk of compromising the method [**1**].

This article reflects the intention of the author to expose another important chapter concerning a series of well-known complications in laparoscopic surgery. In a previous article, we have presented the hemorrhagic complications that occur in our activity. This time we are trying to evaluate another type of complication that survey following celioscopic procedures, sometimes having an unexpected and difficult evolution, that of the post-operatory peritonitis. 

We remind that, as a principle, this complication is the consequence of a series of intra-operatory incidents and accidents (acknowledged or missed), or to a defective laparoscopic technique, all these concurring inevitably to some intra-operatory inadvertences. 

In rare cases, peritonitis post laparoscopic procedures are a direct result of perforator incidences of the intra abdominal organs that usually take place during the procedure to enter in the peritoneum. These incidents usually take place in the beginning of the surgery, are conspicuous, have an important psychological impact, and, in some situations, can influence the progression of the operatory procedure [**2**]. In this situation, the recognition and the immediate solving of the perforator incident are regarded as a primordial condition, but not sufficient, for a favorable post operatory evolution. 

For these reasons, avoiding the visceral lesions, we prefer to introduce the first trocar, using the open method (Hasson) [**3**]. 

The majority of these types of intra-peritoneal complications are the consequence of unknown intra-operator lesions or defective laparoscopic techniques; they can manifest from a few hours to months from the primary operation and can induce a non-favorable evolution.

### History

Even though the advantages of the minimally invasive procedure are undisputed, by reducing the incidence of the post-operatory complications, we acknowledge the fact that sometimes, the method itself can create problems. For this reason, when choosing a laparoscopic procedure all its risks must be taken into consideration [**4,5**].

Evidence that this subject is not at all neglected are the various statistics we find in the specialty literature, lots of publications that present the post-operatory peritonitis after laparoscopic surgery as a complication. 

Starting with the classification concerning different difficulty grades of the early post-operatory complications, put forward by Clavien in 1992 [**6**] and afterwards revised in 2009 by Clavien- Dindo [**7**], a series of foreign authors published their results. 

In 2000, American specialists had encouraging perspectives concerning laparoscopic reinterventions imposed by post-operatory peritonitis following minimally invasive surgery. This was a sign that this problem was long in their attention and activity [**8,9**].

In the same year, Wittgrove and Clark published a statistic of post laparoscopic surgery peritonitis that presents a 3% rate [**10**].

A 2002 Australian study presented the fact that only half of the iatrogenic lesions of the bilious duct are noticed during surgery [**11**].

In 2006, specialists from South America published a 2.8% rate of the intra peritoneal abscesses following laparoscopic appendectomy. The study was published on a statistic of 3433 cases [**12**].

Following a retrospective study concerning 21 laparoscopic operated cases of colorectal cancer, Italian surgeons published in 2008 a zero rate of morbidity and mortality concerning post-operatory peritonitis [**13**].

Finally, 2011 came with new surprise concerning laparoscopy. Until now, the single progress of the laparoscopic technique conferred us additional safety during complicated celioscopic procedures. Today we realize that the miraculous instrument that can confer us absolute safety during surgery has not been invented yet. This is the conclusion reached by famous Leroy and Marescaux. They presented a case of purulent peritonitis as a consequence of a thermal lateral colonic injury produced by a Ligasure forceps [**14**].

### Objectives

The purpose of our study is to find out the answers to some pertinent questions concerning the severity of post-laparoscopic peritonitis (the morbidity and mortality rate of this pathology), the mechanics that triggers the process, the impact of a complicated evolution due to peritonitis in the case of patients who had laparoscopic bariatric surgery, and last but not least, if this complications can be resolved by performing a laparoscopic reintervention?

We had an accurate follow up of the evolution in patients subjected to bariatric surgery who had post-operatory peritonitis as an early or late complication.

Also, as a purpose of our study, we screened the utility of laparoscopic reintervention as a method for diagnosis and solving some of the peritoneal post-operatory complications.

## Material and method 

We have concluded the retrospective analyses, of the last decade, based upon the laparoscopic experience of the Clinic of General Surgery, Emergency Hospital “Sf. Ioan”, Bucharest (01.01.2003-31.12.2010). Out of 18676 laparoscopic procedures, we recorded 106 (0.57%) post-operatory peritonitis that were subjected to 180 (0.96%) laparoscopic reintervention surgery. The rest of the complicated cases required classical surgery. 

We established a series of criteria that allowed us to select the cases included in the study. First, we selected all the patients who had complications that imposed reintervention (gravity grade II, III, and IV Clavien-Dindo) [**7,15**].

In the end, we included in the study the patient who’s certain or probabilistic peritonitis complications imposed early classical or laparoscopic surgery. In addition, we added in, the patients who had an atypical post-operatory evolution that required another laparoscopy to establish the diagnosis.

In a special part of this study, we have a recent analysis of laparoscopic bariatric interventions made in our clinic, an exceptional acquisition in last 10 years. The 2150 interventions were 1430 cases of Laparoscopic Sleeve Gastrectomy (LSG), 250 Laparoscopic Gastric Banding (LGB) and 470 Laparoscopic Gastric By-pass (LGBY). I 16 cases with postoperative peritonitis solved by reinterventions. 

Most of the reinterventions were not exclusively inputted to the laparoscopic type of procedure required for this pathology [**16**]. In addition, the majority of the diagnostic elements suggesting peritonitis evaluated by the surgeons were omitted by the paraclinical examinations (ultrasound, CT scan) [**17**].

The cases that did not require reintervention were not taken into consideration. The treatment consisted in conservatory antibiotic therapy.

The examination charts and the operatory protocols, of all the patients with laparoscopic reintervention procedures in the beginning that were concluded in open surgery, were analyzed. In addition, we reviewed the examination charts of the patients readmitted for various pathologies following a laparoscopic surgery.

## Results

**Table 1:** Laparoscopic practice of the General Surgery Clinic, Emergency Hospital “Sf. Ioan” Bucharest (2000-2010)

Year	Total laparoscopies	Reintervention after laparoscopies	Reintervention for peritonitis	Peritonitis after appendectomy	Peritonitis after cholecystectomy	Peritonitis after bariatric surgery	Peritonitis after other laparoscopy
2000	859	10	3	0	1	0	2
2001	1014	10	6	2	2	0	2
2002	1621	5	2	1	0	0	1
2003	1256	7	4	2	2	0	2
2004	1456	32	15	6	7	0	2
2005	1594	16	14	4	4	5	1
2006	1939	18	11	3	2	3	3
2007	1890	19	13	5	5	1	2
2008	2638	22	14	6	4	2	2
2009	2182	20	12	5	5	2	0
2010	2227	21	12	5	4	3	0
TOTAL	18676	180 (0,96%)	106 (0,57%)	30 (0,20%)	36 (0,19%)	16 (0,74%)*	15 (0,08%)
* % by 2150 of bariatric laparoscopic interventions

As expected, the incidence of post-operatory peritonitis was higher in the cases of laparoscopic appendectomy (0.2%) and cholecystectomy (0.19%). A particular case has been noted among the obese patients who were subjected to laparoscopic sleeve gastrectomy (LSG). A laparoscopic reintervention had to be performed, at one-year distance from the primary procedure, because of a left sub-frenic abscess produced by the rejection of the strip from the gastrectomy trance. 

The morbidity rate by postoperative peritonitis in bariatric surgery is significantly higher than the morbidity rate for the rest of the laparoscopically approached pathology.

Among other laparoscopic procedures resulted in postoperative peritonitis we can mention: antireflux fitting, hysterectomy, colorectal interventions, adrenalectomy, etc.

The graphics revealed the reinterventions incidence and the annual rate of this. There is a pick in the middle of the study period (Graphics **1,2**). This can be explained because that moment corresponds with the time of implementing a new celioscopic procedure (bariatric or colorectal surgery).

**Graphic 1:**
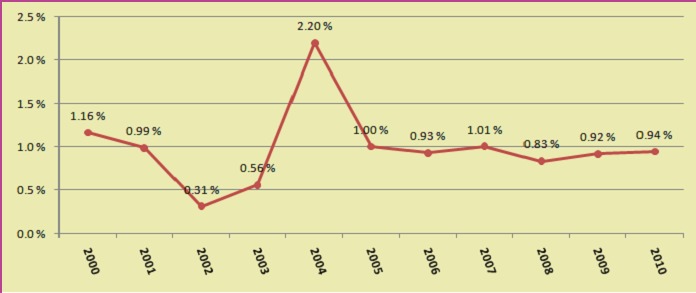
Reintervention incidence after laparoscopies

**Graphic 2:**
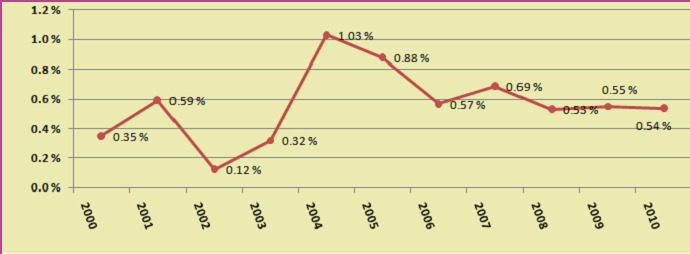
Annual rate of reinterventions after laparoscopies

The rate of reinterventions claimed by the laparoscopic procedures in general and bariatric procedures in special is represented in graphics **3** and **4**

**Graphic 3:**
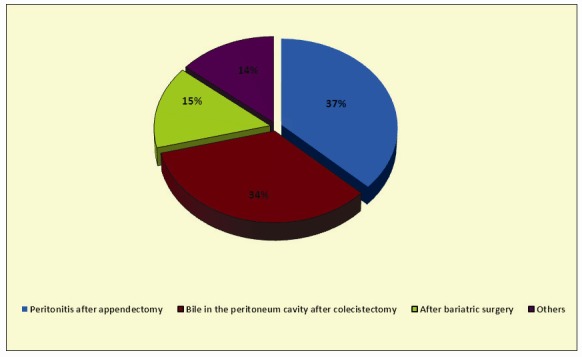
Reinterventions for peritonitis after laparoscopic surgery

**Graphic 4:**
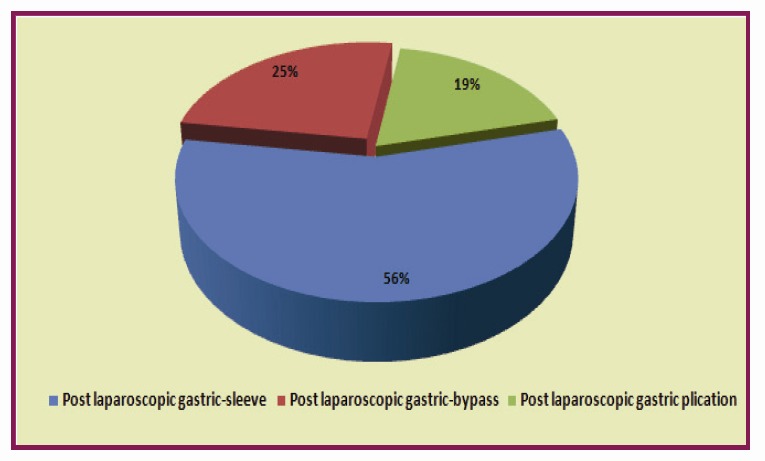
Peritonitis after bariatric laparoscopic surgery

**Fig. 1:**
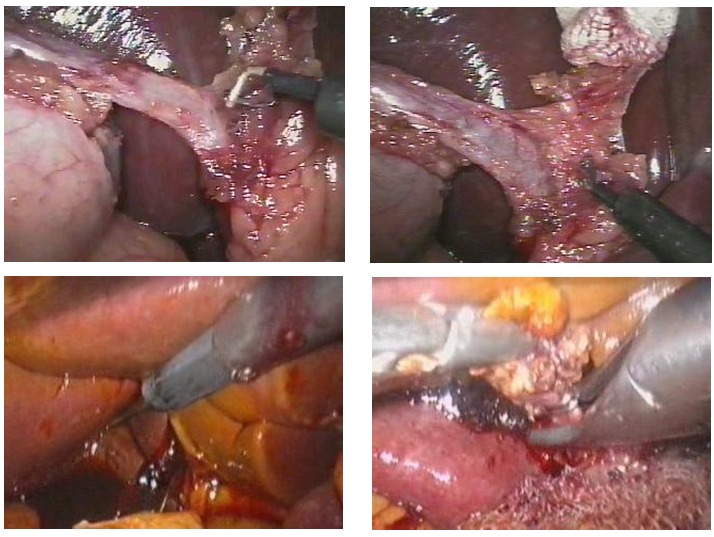
Bile peritonitis upon necrosis of the cystic patch after laparoscopic anterograde cholecystectomy

The important thing about biliary peritonitis (**Fig. 1**) is finding out the probable cause, taking into consideration the fact that surgery usually occurs without intra-operative incidents. The scenario for such a complication can probably be built on the excessive dissection of the cystic channel or the use of inappropriate currents, errors that would generate the electrical injure of the bile wall and the postoperative detachment of the bedsores.

**Fig. 2:**
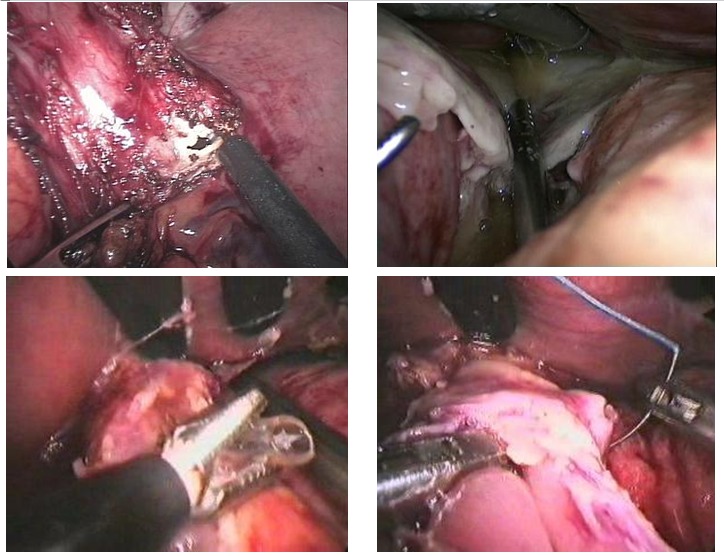
Purulent peritonitis consecutive to Floppy-Nissen laparoscopic procedure

**Fig. 3:**
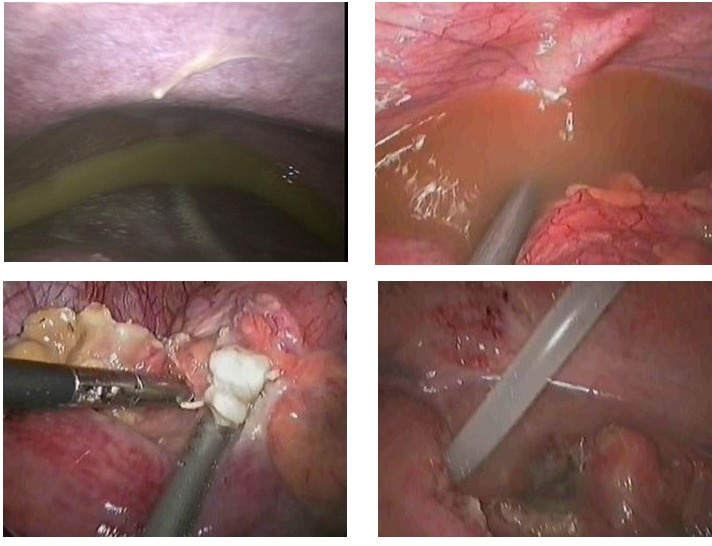
Purulent general peritonitis after laparoscopic appendectomy

The intervention was performed for acute gangrenous appendicitis. The cause of this complication has proved to be the amputations of the apendicular remain in the area of the ligation (**Fig. 3**). In the absence of a continuity solution for the digestive lumen, the chosen method of solving the complication was multiple lavage and drainage of the peritoneal cavity, completed with the neighboring drainage of the apendicular remain.

**Fig. 4:**
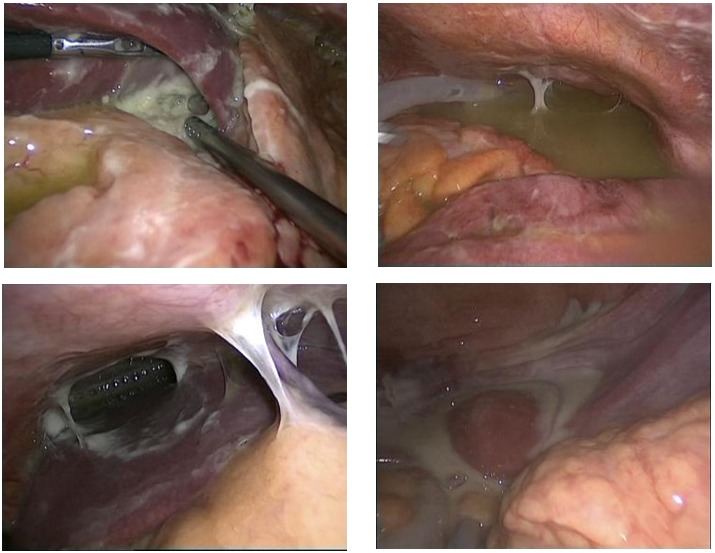
Purulent general peritonitis after Laparoscopic gastric Sleeve

The severity of the peritonitis occurred after a bariatric intervention is already known to those practicing this type of surgery (**Fig. 4**). In the case of patients with morbid obesity, the complications become serious, given their biological fragility and the rapid installation of hemodynamic imbalances under septic shock [**18,19**]. This is the reason why bariatric surgeons have lately adopted a firm attitude of emergency precocious laparoscopic reintervention every time there is suspicion of intra peritoneal septic process, consecutive to a more or less recent bariatric surgery [**20,21**]. 

The extreme inflammatory tissue modifications in the peritoneal cavity, installed at the same time with the occurrence of these complications, determined us to convert laparoscopy to the classical method in 5 out of 15 reinterventions for post-operatory peritonitis.

**Fig. 5:**
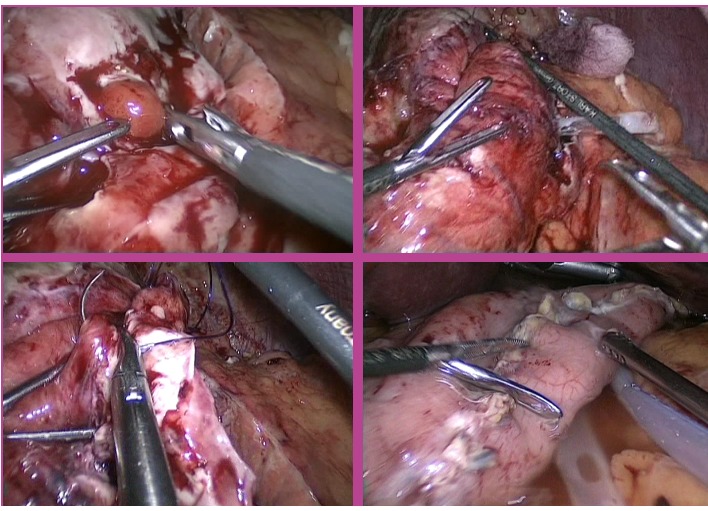
Laparoscopic approach of post-operatory peritonitis

The solutions for the surgical solving have been adapted to each particular case. In 5 out of 16 reinterventions performed for postoperative peritonitis, laparoscopy has proved inadequate for treating the cause, the conversion being necessary. The laparoscopic reinterventions will be finalized after a rigorous control of the entire peritoneal cavity, to be certain of the complete solving of the deficiencies from the primary surgery. It is also important to keep in mind the fact that through laparoscopy both heavy repeated lavage of the peritoneal cavity, for the removal of the accumulated pus and the multiple peritoneal drainage similar to the open one, but having the advantage of preserving the integrity of the abdominal wall, are possible (**Fig. 5**).

## Discussions

Currently, there is a profound change in the perception of medical activity on behalf of the public opinion. Not a long time ago, the surgeon was regarded as a benefactor who was allowed to commit certain errors (even if seldom). Nowadays, he is perceived as a professional who implicitly works according to a contract that stipulates the possibility of legal action from the patient, when he is not satisfied with the result [**22**].

Postoperative peritonitis, a possible cause of malpraxis, is the consequence of incidents and accidents not recognized intraoperatively or of a faulty maneuvering of the laparoscopic instruments (Ligasure forceps, steppler Endogia, morselator).

A key element in the occurrence of such inadvertences is also represented by the technical factor associated to certain moments, especially at the beginning of the laparoscopic activity. 

The lesions responsible for the occurrence of post-operative peritonitis most frequently reside in the creation of the laparoscopic working field (creation of the pneumoperitoneum, insertion of trocars), maneuvering of the instruments outside the visual field (electrical escars, mechanical lesions), the use of technically improper instruments, the anatomically intra-operative not recognized versions, the extraction of the biliary drainage catheter. 

In our study, the postoperative mortality rate through peritonitis consequent to laparoscopic interventions was of 0.04% (8 out of 18676 cases).

The mortality rate through this type of complication, occurred following laparoscopic bariatric surgery, is of 0.18%, our results being in accordance with other recent statistics presented in the specialty literature (0.1-2%) [**23-25**]. The experience gained in the last 10 years of laparoscopic activity entitles us to state that the onset for this type of intervention has been surpassed, the premises for an ample development in this direction already existing [**26**].

With regard to the bariatric laparoscopic interventions, evidence has been provided of an inversely relation between the experience of the surgeon and the mortality/morbidity rate [**27,28**].

## Conclusions

In laparoscopic surgery, postoperative peritonitis is inevitable and imposes a firm attitude concerning the lowering of intraoperative risks but particularly in choosing the right therapeutically conduct. 

The celioscopic method has proved useful also in dealing with this type of complication. 

Taking into consideration the generated mortality and morbidity, post-operative peritonitis is situated among the most severe complications of minimally invasive surgery. Such cases can be solved through laparoscopic reinterventions only with help from an extremely experienced medical team and under impeccable technical conditions. The share of this type of severe complication remains low, with no perspective of compromising the celioscopic method overall.
